# Application of joint modeling on the determinants of TB Status and CD4 cell count among antiretroviral therapy attendants in Gondar teaching referral hospital, Gonder, Ethiopia

**DOI:** 10.4314/ahs.v23i4.2

**Published:** 2023-12

**Authors:** Kindu Kebede Gebre, Nuru Mohammed Hussen

**Affiliations:** 1 Department of Statistics, Haramaya University, Dire Dawa, Ethiopia; 2 Department of Statistics, Samara University, Semera, Ethiopia

**Keywords:** CD4 count, Co-infection, Tuberculosis, Joint model

## Abstract

**Background:**

East African regions were highly affected by tuberculosis and the human immunodeficiency virus. The main objective was to identifying the associated factors with tuberculosis and CD4 cell count of patients in Gonder teaching referral hospital, Gonder, Ethiopia.

**Methods:**

A retrospective cohort study was conducted on AIDS patients from 1^st^ January 2018 - to 30^th^ January 2020. This study used joint mixed model, and individual profile plot to identify factors and the changeability inside and between patients respectively.

**Results:**

The mean with a standard deviation of weight and a serum hemoglobin concentration of patients were 55.48 (10.21) kilograms and 18.25 (33.028) grams per decilitre respectively.

This study shows an opportunistic infection, weight, and serum hemoglobin concentration were significantly associated with the log CD4 cell count and tuberculosis status of patients.

**Conclusion:**

The patient who has other diseases is 5.04 more likely to be co-infected with HIV and TB diseases. And also, the estimated odds of being co-infected in both diseases were increased by 1.14 and 1.05 times when a unit change in weight and hemoglobin respectively. Moreover, the estimated odd of patients who have no other related disease were 51.13% less likely to be co-infected with both diseases.

## Introduction

Human Immune Deficiency Virus is a virus that causes acquired immune deficiency syndrome by reducing a person's ability to fight infection. HIV attacks an immune cell and the CD4 cell is responsible for the body's immune response to infectious agents. HIV is associated with dramatic declines in morbidity and mortality as a result of highly active antiretroviral therapy, and treatment response rates with subsequent antiretroviral regimens are lower than with initial antiretroviral therapy 1. East and Southern Africa regions were highly affected by HIV. East and Southern Africa regions are home to 6.2% of the world's population, but they have 19.4 million people living with the virus, over 50% of the total number of people living with the virus in the world 2.

HIV positive people have high hazard to create TB as compared to HIV-negative people. Out of the 8.8 million occurrences of TB cases around the world, 1.1 million were found to be co-infected with HIV 3.

Tuberculosis is a leading opportunistic infection and a major cause of mortality among individuals infected with HIV. Substantial reduction of tuberculosis-related morbidity and mortality among individuals with HIV can be achieved with early initiation of ART 4.

HIV/AIDS pandemic is responsible for the resurgence of TB worldwide, resulting in increased morbidity and mortality. Co-infection with HIV infection leads to difficulties in both the diagnosis and treatment of tuberculosis, increase risk of death, treatment failure, and relapse 5. Today, HIV and TB treatments are common in many societies and the use of drugs has altered the joint dynamics of both diseases. About one third of 39.5 million HIV infected people worldwide were co-infected with TB 6 and up to 50 % of individuals living with HIV are expected to develop TB 7.

Ethiopia is among the countries most heavily affected by the Human immunodeficiency Virus and tuberculosis. There are an estimated 1.3 million people living with the virus and roughly 68,136 of them were children under 15 years. World Health Organization has classified Ethiopia as the 7th among the 22 high burden countries with TB and HIV infection in the world 8.

As stated in the literature, many studies conducted in lined to tuberculosis and HIV co-infection related in Ethiopia were mainly focused on the knowledge of health providers about tuberculosis and HIV co-infection 8, tuberculosis in HIV/AIDS patients and its relationship with CD4 count 5, tuberculosis and HIV co-infected patients 4. In spite of the fact that, several studies have been done on assessment and examined general tuberculosis and HIV co-infection, they didn't deal with the joint determinants of tuberculosis status and CD4 cell count using joint modeling.

Several studies have been conducted with separate analysis for the two outcomes that were fitting a logistic model for the TB status of patients and a Poisson regression model for the CD4 count. However, separate analyses of the two outcomes ignore the correlation between the two outcomes.

In order to fill the gap, in many medical cases more than one clinical outcome is measured longitudinally at the same time on the same subject where these measured clinical outcomes are correlated. Since they are highly related changes in either of them often affects changes in the other. In such cases the univariate longitudinal analysis does not take into account the correlation between observations on different response variables at each time points. Besides, joint modeling of longitudinal data in other way round accounts two types of correlations which are known to be serial correlation and cross correlation 9. The response variables are bivariate outcome such as CD4 count and TB status of patients. This study is concerned with joint modeling of two outcomes which is account two types of correlations which are known to be serial correlation and cross correlation. In the joint model data sets must be changed from multivariate form to univariate form. In the multivariate form the responses are stored in separate variables. The current study was targeted on identifying the common determinants of tuberculosis and CD4 cell count of patients. The data step in this study expands the observations in the data set into observations, stacking two observations per patient. The character variables identify the distribution that is assumed for the respective observations within a patient. But univariate longitudinal analysis does not take into account of the correlation between observations on different response variables at each time points. This study considered both responses jointly to consider the correlation between observations on different response variables at each time points.

The study used the joint model, which is more important to control errors, especially type I error rates, and has greater efficiency in parameter estimation.

## Methods

### Data source and study design

This study was conducted at Gondar Teaching Referral Hospital in North-Western Ethiopia, Amhara Region. The target population of this study included HIV positive individuals who attend antiretroviral therapy at the study area. Moreover, HIV positive patients who were greater than 19 years old and have started ART since January 1, 2018, and who has a base line at least three follow up the period until January 30, 2020, were included in this study. This study obtained data from a retrospective cohort study based on ART electronic data base and from the review of patient charts which contains socio-demographic, laboratory and clinical information of all patients under ART follow-up including a detailed antiretroviral therapy history during the follow-up times. CD4 cell level and TB status of the patients were collected at the initiation of the treatment and at different time points after the start of the treatment. Subjects come to the center at irregular time (one, two three or more month's gap) and visit their CD4 cell level is measured and recorded in the individual follow up cards.

### Variables included in the study

The response variables for this study were CD4 cell count which is a count variable and TB screen results (positive or negative) which is the binary outcome of AIDS patients. The predictor variables that are included in this study were background characteristics of AIDS patients and history of epidemiological, clinical, and the laboratory results (Table 1). Data were entered and cleaned using SAS version 9.4.

**Table 1:** Study variables

Gender	0=Female, 1= male
Age in years	Continuous
Marital status	1=single,2= married,3=divorced,4=widowed
Weight	Continuous in Kg
Adherence status	1=good,2=fair,3=poor
WHO Clinic Stage	1= Stage I,2= Stage II,3= Stage III,4= Stage IV
Functional status	1=working,2=ambulatory,3=bedridden
Hemoglobin level	Continuous variable in g/dl
TB screening	1=Negative, 2= Positive
Duration of ART	Count
Baseline CD4	Count
Religion	1= Orthodox, 2= Muslim, 3=Protestant and 4= other
Educational level	0= No education,1=Primary,2=Secondary,3= Tertiary
occupation	0=Government,1=Farmer,2=Ngo,3=Self-worker,4=Housewife,5=Unemployment, 6= Other
Opportunistic infection status	1= yes, 0=no
Regimen	0=d4t-3TC-NVP,1=d4t-3TC-EFV,2=AZT-3TC-NVP,3=AZT-3TC-EFV,4=TDF-3TC-EFV,5=TDF+3TC+NVP

### Statistical analysis technique

Longitudinal data have multiple observations to the same attribute measured at different points in time. This leads to repeated measures which are special forms of multivariate data. A different class of multivariate data arises when the multiple observations refer to different to assess the changes of outcome (s) over time to associated risk factors with the bivariate outcome variable by using joint modeling of binary and count data 10. There are several strategies for adopting joint modeling. The first approach is based on a conditioning argument that allows joint distribution to be factor out in marginal and conditional components (avoiding direct specification of joint modeling) with introduction of probit approach. This approach has advantages that it does not directly lead to marginal inference and the correlation between the two outcomes can't be directly estimated. The second is direct formulation of joint modeling for both response variables with the introduction of placket –Dale approach (placket latent variable) assumption for modeling bivariate outcomes. To obtain valid inferences, joint models could account for the correction among the outcomes and effects of different factors. The joint model assumes it for each outcome, and the univariate models are combined through specification of joint multivariate distribution for allandom effects. Furthermore, the joint model can be applied with specification of marginal distribution, conditional on correlated random effect. When we have cases that, there are many outcomes measured from the longitudinal data, the most basic approach would be to model each longitudinal response independently.

Let ^*Y*^*kij* = (*Y*^T^*ki*1, *Y*^T^*ki*2,..*Y^T^kim*)^T^ be the k^th^ outcome for i^th^ subject at j^th^ measurement (j = 1, 2,…,^n^*t*). For bivariate response cases, we have k=1, 2 and two independent repeated measure equations be given as;

*Y*1*ij* = (*Y*1*i*1, *Y*1*i*2,… *Y*1*int*)^T^ is the first outcome for i^th^ individual at the j^th^ measurement and

*Y*2*ij* = (*Y*2i1, *Y*2*i*2,…, *Y*2*int*)^T^ is the second outcome for i^th^ individual at the j^th^ measurement

Assume that *^Y^*1*ij* (j = 1, 2,… *nt*1 and *i*= 1,2,…,*n* are conditionally independent given that ^V^1*i* with density *f*_1_ (.) and the link function *h*_1_(.) in the exponential family. Also let *Y*2*ij* (j = 1,2,…,*ni*2 are conditionally independent given that ^V^2*i* with density *f*_2_ (.) and link function *h*_2_ (.) in the exponential family. Also, *Y*1*t* and *Y*2*t* are conditionally independent given ^V^*t*= (*Y*1*t Y*2*i*)^T^ and responses on different subjects are independent.

Let *N*1*tj* and *N*2*ij* be the conditional mean of *Y*1*tj* and *Y*2*tj* respectively such that

*N*1*tj* = (*N*1*i*1, *N*1*i*2,,,*N*1*ini*1)^T^ and

*N*2*tj* = (*N*2*i*1, *N*2*i*2,,,*N*2*ini*2)^T^

The marginal covariance matrix between *Y*1*t* and *Y*2*t* is found to be equal to the covariance between N1i and N2i that is Cov (Y1*t*, Y2*t*) = cov (*N*1*i*, N2*t*). This property is a consequence of the key assumption of conditional independence between the two responses. It allows an extension of model fitting methods from the univariate to the multivariate GLMM 12.

### Parameter estimation

This study conducts parameter estimation for joint models, which follows the following steps: First, joint marginal models will be fitted for both responses, and then the linearization estimation method will be used as an approximation method. The parameters of joint models can be estimated using numerical approximation methods. These include approximations to the integral using Gaussian quadrature or the Laplace approximation 13. The other estimation method is based on an approximation of the data using the pseudo-likelihood, in which pseudo-data are created based on a linearization of the mean. More specifically, the pseudo-likelihood approach can be used to estimate parameters in marginal models and random effects with or without serial correlation, whereas quadrature or Laplace approximation can only estimate parameters in conditional independent random effect models.

### Missing data treatment

Missing values are a common issue in a lot of practical data situations. There are many imputation methods for handling missing values in a longitudinal study. However, the most popular imputation method for handling missing values is multiple imputations 14. This study used the multiple imputation method.

## Results

A total of 1408 AIDS patients were included in the study. From summarized in Table 2 shows that the most participant in this study was female (61.2%). From marital status point of view 47.9% were married and 6.3% were widowed. In addition, the highest and the lowest participants (76.1%) and (3.4%) who follow orthodox and protestant religion respectively. In this study 97.6% patients who have no other infectious disease rather than AIDS and TB. Regarding the education level 49.3% patients had tertiary education level, 28.7% patients had secondary education level and 4.1% patients were illiterate.

**Table 2:** Summary of socio-demographic covariates that associated to TB skin test of patients

Variables	Levels	TB Skin Test				
		Negative		Positive		
		Count	%	Count	%	Total (%)
Gender	Female	107	7.6	753	53.5	860(61.2)
	Male	71	5.0	477	33.9	548(38.8)
Marital status	Single	26	1.8	191	13.6	217(15.5)
	Married	79	5.6	589	41.8	668(47.9)
	Divorced	57	4.0	372	26.4	429(30.2)
	Widowed	16	1.1	78	5.5	94(6.3)
Religion	Orthodox	142	10.1	936	66.5	1078(76.1)
	Muslim	22	1.6	175	12.4	197(14.2)
	Protestant	3	.2	42	3.0	45(3.4)
	Other	11	.8	77	5.5	88(6.3)
Education	No education	10	.7	51	3.6	61(4.1)
	Primary	38	2.7	220	15.6	258(17.9)
	Secondary	43	3.1	353	25.1	396(28.7)
	Tertiary	87	6.2	606	43.0	693(49.3)
Occupation	Governmental	25	1.8	258	18.3	2283(21.0)
	Farmer	14	1.0	73	5.2	87(5.9)
	NGO	10	.7	52	3.7	62(4.2)
	Self-Worker	39	2.8	289	20.5	328(23.5)
	House-wife	40	2.8	198	14.1	238(16.1)
	Unemployment	16	1.1	90	6.4	106(7.3)
	Other	34	2.4	270	19.2	304(22.0)
Functional status	Working	115	8.2	1160	82.4	1275(94.3)
	Ambulatory	27	1.9	62	4.4	89(5.0)
	bedridden	36	2.6	8	0.6	44(0.7)
WHO stage	Stage I	117	8.5	1073	77.9	1190(87.2)
	Stage II	16	1.2	92	6.7	108(7.5)
	Stage III	11	0.8	56	4.1	67(4.6)
	Stage IV	3	0.2	9	0.7	12(0.7)
Opportunistic infection status Adherence status	Yes	20	1.4	29	2.1	49(2.4)
	No	158	11.2	1201	85.3	1359(97.6)
	Good	103	7.3	1094	77.7	1197(88.9)
	Fair	10	0.7	18	1.3	28(1.5)
	Poor	65	4.6	118	8.4	183(9.6)

Among all the AIDS patients studied 53.5% live with TB, and 94.3% had working functional status in the first-month treatment. Over 87.2% of them had WHO stage one. Out of 860 (61.2%) female AIDS patients 753 (53.5%) were lived with TB while 107 (7.6%) of them have no TB.

Out of 668 (47.9%) married AIDS patients, 589 (41.8%) were lived with TB while 79 (5.6%) of them were free from TB. From total 693 (49.3%) AIDS patients who have tertiary educational level and 606 (43%) were lived with TB. Moreover, out of 328 (23.5%) self-workers of occupation, 289 (20.5) were lived with TB. And also, from total 1275 (94.3%) working functional status group of AIDS patients 1160 (82.4%) were lived with TB. Finally, from total 1197 (88.9%) good adherence status of AIDS patients, 1094 (77.7) were lived with TB.

The descriptive statistics of continues variables are summarized in Table 3. Out of the total 1408 patients were included in the study; the mean of weight of patients is 57.2 kg with standard deviation 10.4 kg, and the average age were 35 years with standard deviation 10 years approximately. In addition, the average baseline CD4 was 293 cells per cubic millimeter with standard deviation 167 cells per cubic millimeter. Moreover, the average CD4 count of patients was 396 cells per cubic millimeter with standard deviation 205 cells per cubic millimeter, and the mean of hemoglobin were 30.2 with standard deviation 83.8. The maximum baseline CD4 cell count for all patients was 980 cells per cubic millimeter of blood respectively.

**Table 3:** Descriptive statistics for continues covariates and outcome variables that included in this study

	Mean	Minimum	Maximum	Standard deviation
Age	35	15	70	10
Weight	57.3	24.9	88	10.4
Baseline CD4	293	65	980	167
CD4 count	396	0	1440	205
Hemoglobin level	30.3	9.1	888	83.8

**Figure 1:**
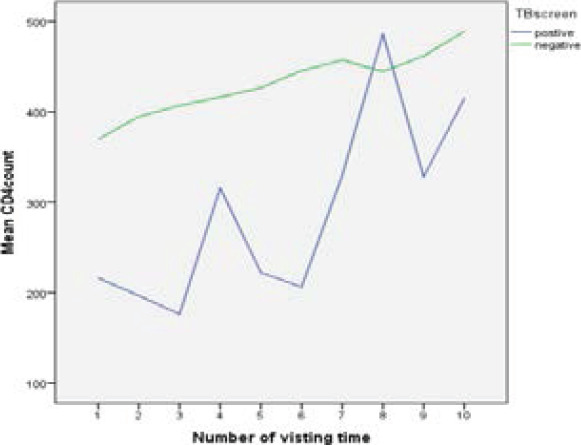
Line graph of CD4 cell of AIDS Patients and TB skin test over their visiting time

**Figure 2:**
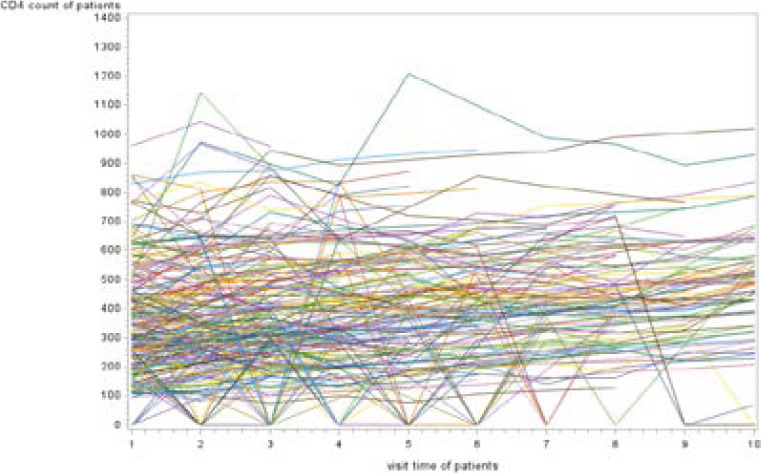
Individual profile plot of AIDS patients

Figure 1 indicated that the likelihood of being infected with TB was slightly increasing on each respondent throughout their follow-up times. For responses, most (but not all) observations were slightly turned down from first to third follow-up times. However, the mean pattern of CD4 count measurements of the patients between sixth and eighth visiting times was increased. The mean of CD4 cell for AIDS patients lived with TB is maximum at the eight-visiting time. However, Figure 2 shows that the changeability inside and between patients was marginally decreasing on each patient throughout the follow-up.

Table 4 contains a separate column for each response distribution, as well as an overall contribution. Because the model does not specify any random effects or R-side correlations, the log likelihoods are additive. The parameter estimates and their standard errors in this joint model are identical. There are two ways in which the correlations between the two responses for the same patient can be incorporated. We can induce them through shared random effects or model the dependency directly. A joint model of the second kind, where correlations are modeled directly, fit with the generalized linear mixed effects model statements. Table 4 indicates the separate or joint marginal models for CD4 cell count change and TB status using Poisson and binary logistic regression. As indicated in the Table 4, time to visit, weight, initial CD4 cell count, serum hemoglobin concentration, education level, religion, marital status, occupation and age of patients significantly affected CD4 cell count change. And also, occupation, opportunistic infection, ART adherence, weight and hemoglobin level of patients significantly affected TB screen outcomes. But weight, serum hemoglobin concentration and occupation of patients were associated to both outcomes significantly at alpha equal to 5%.

**Table 4:** Parameter estimation of separate models

Parameter	Estimate	CD4 count	TB status		

		Std. Error	P. value	Estimate	P. value
(Intercept)	4.566	2.0210E-015	.000	2.1357	.756
[time=1]	-.163	.0042	.000	.6695	.705
[time=2]	-.138	.0003	.000	.6655	.416
[time=3]	-.116^a^	.	.	.6948	.892
[time=4]	-.086	.0033	.000	.6761	.155
[time=5]	-.079	.0054	.000	.6887	.123
[time=6]	-.052	.0053	.000	.7851	.768
[time=7]	-.044	.0059	.000	.7068	.140
[time=8]	-.040	.0060	.000	.7667	.559
[time=9]	.010	.0068	.138	.8799	.996
[time=10]	0^b^	.	.	.	.
[Gender=0]	.008^a^	.	.	.2587	.776
[Gender=1]	0^b^	.	.	.	.
[Marital status=1]	-.059	.0040	.000	.5901	.369
[Marital status=2]	.040^a^	.	.	.5226	.892
[Marital status=3]	-.016	.0031	.000	.5311	.860
[Marital status=4]	0^b^	.	.	.	.
[Religion=1]	-.350^a^	.	.	.4673	.641
[Religion=2]	-.423	.0033	.000	.5535	.786
[Religion=3]	-.238	.0089	.000	1.1549	.508
[Religion=4]	0^b^	.	.	.	.
[education=0]	-.338	.0091	.000	.6290	.859
[education=1]	-.020^a^	.	.	.3544	.794
[education=2]	-.054	.0027	.000	.3169	.931
[education=3]	0^b^	.	.	.	.
[Occupation=0]	-.019	.0038	.000	.4369	.800
[Occupation=1]	.130	.0061	.000	.5688	.501
[Occupation=2]	.005	.0071	.479	.5708	.682
[Occupation=3]	.013	.0033	.000	.3840	.487
[Occupation=4]	-.060^a^	.	.	.3867	.020
[Occupation=5]	-.149	.0064	.000	.4830	.317
[Occupation=6]	0^b^	.	.	.	.
[Functional status=1]	.064^a^	.	.	.9555	.226
[Functional status=2]	.339	-1.576	2.255	.9772	.728
[Functional status=3]	0^a^	.	.	.	.
[WHO stage=1]	-.708	-2.499	1.082	.9136	.438
[WHO stage=2]	-.233	-2.145	1.678	.9755	.811
[WHO stage=3]	.122	-1.798	2.042	.9796	.901
[WHO stage=4]	0^a^	.	.	.	.
[OI=1]	1.798	.989	2.608	.4129	.000
[OI=2]	0^a^	.	.	.	.
[ART adherence=1]	-2.425	-4.348	-.501	.9813	.013
[ART adherence=2]	-.176	-2.292	1.940	1.0796	.871
[ART adherence=3]	0^a^	.	.	.	.
[Regimen=0]	2.498	-.160	5.155	1.3559	.065
[Regimen=1]	2.688	-.359	5.735	1.5546	.084
[Regimen=2]	.169	-1.737	2.075	.9725	.862
[Regimen=3]	-.659	-2.631	1.313	1.0061	.513
[Regimen=4]	.226	-1.577	2.030	.9202	.806
[Regimen=5]	0^a^	.	.	.	.
Age	.020	-.005	.044	.0123	.613
weight	.003	-.019	.025	.0113	.781
BaseCD4	.000	-.001	.002	.0007	.760
Hgb	.004	-.016	.024	.0104	.02

The estimate of the variance of the random intercept is 0.1166, and the estimated standard error of this variance component estimate is 0.01301. This result showed that there was a significant variation on the baseline CD4 count of AIDS patients. The estimates of the fixed effects as well as their estimated standard errors have changed from the bivariate-independence analysis (see Table 5). When the CD4 count change and the TB status are modeled jointly and compare the result with the separate analyses. Based on generalized chi square/df value equal to 7.53 is small and closed to one that shows the model is good fit.

**Table 5:** Parameter estimation of random effect model

		Covariance parameter		Fit Statistics		

	Subject	Estimate	Standard Error	2 Res Log Pseudo-Likelihood	Generalized Chi-Square	Gener. Chi-Square / DF
CovParm intercept	id	0.1166	0.01301	20215.70	17927.47	7.53

Table 6 shows the conditional independence random intercepts the model. As indicated patients' serum hemoglobin concentration, weight, and opportunistic infection of other disease were significantly associated with both response variables jointly. The same sign in parametric estimation indicates that the two outcomes are positively correlated to each other. Since the conditional independence assumption might be too restrictive.

**Table 6:** Parameter estimation of joint model

Parameter	CD4count	TB status			

	Estimate	Std. Error	P. value	Estimate	P. value
[Marital status=1]	-.059	.0040	.000	.5901	.369
[Marital status=2]	.040^a^	.	.	.5226	.892
[Marital status=3]	-.016	.0031	.000	.5311	.860
[Marital status=4]	0^b^	.	.	.	.
[Religion=1]	-.350^a^	.	.	.4673	.641
[Religion=2]	-.423	.0033	.000	.5535	.786
[Religion=3]	-.238	.0089	.000	1.1549	.508
[Religion=4]	0^b^	.	.	.	.
[education=0]	-.338	.0091	.000	.6290	.859
[education=1]	-.020^a^	.	.	.3544	.794
[education=2]	-.054	.0027	.000	.3169	.931
[education=3]	0^b^	.	.	.	.
[Occupation=0]	-.019	.0038	.000	.4369	.800
[Occupation=1]	.130	.0061	.000	.5688	.501
[Occupation=2]	.005	.0071	.479	.5708	.682
[Occupation=3]	.013	.0033	.000	.3840	.487
[Occupation=4]	-.060^a^	.	.	.3867	.020
[Occupation=5]	-.149	.0064	.000	.4830	.317
[Occupation=6]	0^b^	.	.	.	.
[OI=1]	1.798	.989	0.018	.4129	.000
[OI=2]	0^a^	.	.	.	.
[ART adherence=1.0]	-2.425	-4.348	.501	.9813	.013
[ART adherence=2.0]	-.176	-2.292	1.940	1.0796	.871
[ART adherence=3.0]	0^a^	.	.	.	.
weight	.003	-.019	.025	.0113	.021
BaseCD4	.000	-.001	.002	.0007	.760
Hgb	.004	-.016	.024	.0104	.02

## Discussion

In this study, two different models were explored, separate models for each outcome independently and joint modeling of the two outcomes together. In the separate analysis of the count and binary data, the log transformation CD4 cells count measurements were used to meet the normality assumption. In this study, the joint modeling of the two outcomes together was well fitted because generalized Chi square/df was 7.53 which closed to one. The results in this study indicated that the separate models which did not include patients' specific effects were not significantly different from joint models developed with the assumption of separate analysis. As indicated above the joint models were formed by imposing the joint multivariate distribution of random effect, Hence, the results of both separate and joint analysis were consistent. However, the joint models were simpler as compared to the separate models as their effective member of parameters was smaller 15-18.

Based on Table 1, time to visit, weight, initial CD4 cell count, hemoglobin level, education level, religion, marital status, occupation and age of patients significantly affected CD4 cell count change and occupation, opportunistic infection, ART adherence, weight and hemoglobin level of patients significantly affected TB screen outcomes. But weight, hemoglobin level and occupation of patients were associated to both outcomes significantly at alpha equal to 5%. This estimated result also consistent with similar previous studies conducted by different scholars 19, 20.

From this study, hemoglobin level, weight, and opportunistic infection of other disease were statistically significant at a 5% level of significance for the log CD4 count and TB status of patients jointly. In addition, the result of the study shows that the log CD4 count of patients increased when hemoglobin level and weight of patients increased. The finding is consistent with 21. Moreover, the log CD4 count of AIDS patients who has other disease is 5.04 more likely to be infected with TB than who has no other disease, controlling other predictors as constant. This result consistent with a previous finding 22.

The estimated odds of being infected with TB were increased by 1.14 and 1.05 for a unit change in weight and hemoglobin respectively. The estimated odds of patients who have no other related disease were 51.13% less likely to be infected with TB as compared to those who have other related disease at 5% level of significant, controlling others predictors constant. This estimated result also consistent with similar previous studies conducted by different scholars 22-27.

## Conclusions

This study was targeted on identifying the joint determinants of CD4 cell count and TB status of HIV AIDS patients attending Gondar teaching referral Hospital from January 1, 2018, to January 30, 2020.Joint analysis of two response variables was assuming their separate analysis. The results in this study indicated that the separate models which did not include patients' specific effects were not significantly different from joint models developed with the assumption of separate analysis. As indicated above the joint models were formed by imposing the joint multivariate distribution of random effect. Hence, the results of both separate and joint analysis were consistent. However, the joint models were simpler as compared to the separate models as their effective member of parameters was smaller.

From this study, hemoglobin level, weight, and opportunistic infection of other disease were statistically significant at a 5% level of significance for the log CD4 count and TB status of patients jointly. In addition, the result of the study shows that the log CD4 count of patients increased when hemoglobin level and weight of patients increased. Moreover, the log CD4 count of AIDS patients who has other disease is 5.04 more likely to be co-infection than who has no other disease, controlling other predictors as constant.

The estimated odd of being infected by TB was increased by 1.14 and 1.05 for a kilogram and gram per deciliters changes in weight and hemoglobin respectively. The estimated odds of patients who have no other related disease were 51.13% less likely to be infected by TB as compared to those who have other related diseases at 5% level of significant, controlling others predictors constant.

## Data Availability

The raw data used in this study can be accessed from the Gondar Teaching Referral Hospital and also the recognized data could also be created accessibly if a unique request.

## List of abbreviation

AIDS: Acquired Immune deficiency Syndrome
ART: Antiretroviral Therapy
CD4: Cluster of Differentiation Four
DF: Degree Freedom
HAART: Highly active antiretroviral therapy
HIV: Human Immunodeficiency Virus
SAS: Statistical analysis system
TB: Tuberculosis
UNAIDS: United Nation Report on Acquired Immune Deficiency Syndrome
WHO: World Health Organization
